# Candy Cane Limb Revision After Roux-en-Y: A Four-Patient Case Series

**DOI:** 10.7759/cureus.87121

**Published:** 2025-07-01

**Authors:** Mena Louis, Jerrell Fang, Jay Narula, Nathaniel Grabill, Eric Velazquez

**Affiliations:** 1 General Surgery, Northeast Georgia Medical Center Gainesville, Gainesville, USA; 2 General Surgery Residency, Northeast Georgia Medical Center Gainesville, Gainesville, USA; 3 Internal Medicine, Northeast Georgia Medical Center Gainesville, Gainesville, USA; 4 Surgery, Northeast Georgia Medical Center Gainesville, Gainesville, USA; 5 Bariatric Surgery, Northside Hospital Duluth, Duluth, USA; 6 Bariatric Surgery, Northeast Georgia Medical Center Gainesville, Gainesville, USA

**Keywords:** anastomotic technique, bariatric complications, blind limb stasis, candy cane syndrome, gastrojejunostomy revision, redundant afferent limb, robotic revisional surgery, roux-en-y gastric bypass

## Abstract

Candy cane syndrome is an uncommon yet clinically significant complication of Roux-en-Y gastric bypass surgery, causing symptoms such as nausea, vomiting, dysphagia, abdominal pain, and persistent gastrointestinal discomfort. These symptoms arise from a redundant afferent Roux limb near the gastrojejunostomy, which causes food stasis and mechanical irritation. Diagnosis can be challenging, as symptoms frequently mimic other common postoperative complications, necessitating careful evaluation through advanced imaging studies and endoscopic assessments. It is most effectively established through upper endoscopy and contrast imaging, both of which can reveal the blind limb segment. When identified, surgical resection of the redundant limb provides a reliable means of symptom resolution. Robotic-assisted revisional surgery facilitates precise dissection and improved visualization, resulting in favorable outcomes. Preventive measures during the initial bypass include appropriate measurement and orientation of the Roux limb and meticulous inspection of the anastomosis.

In this case series, we present four patients diagnosed with candy cane syndrome, illustrating a spectrum of clinical presentations, diagnostic approaches, and surgical revisions. All four patients underwent robotic-assisted revisional surgery that involved precise resection of the redundant Roux limb with reconstruction of the gastrojejunal anastomosis. Postoperative follow-up demonstrated complete resolution of symptoms, significant improvement in quality of life, and no recurrence of symptoms at the time of manuscript preparation.

## Introduction

Roux-en-Y gastric bypass (RYGB) is widely performed for durable weight loss and metabolic improvement, yet the alteration in anatomy caused by the surgery introduces a spectrum of late complications [1]. Patients can present months or even years after surgery with post-prandial pain, nausea, reflux, or unexplained weight change, prompting an extensive differential that includes marginal ulcer, anastomotic stricture, internal hernia, and functional disorders [2]. Among these entities, a redundant afferent Roux limb, commonly called the candy cane syndrome (CCS), has received increasing attention for its capacity to mimic more familiar problems while eluding early diagnosis [3].

The construction of the Roux limb during gastric bypass surgery can vary in technique, but precision is essential to avoid complications such as the formation of a redundant limb [4]. The jejunum is typically divided 30-50 cm distal to the ligament of Treitz to create the biliopancreatic limb, and the alimentary (Roux) limb is measured to an appropriate length before being anastomosed to the gastric pouch [5]. A common technical error occurs when an excessively long blind afferent limb segment is left proximal to the gastrojejunostomy (GJ) [6]. This segment, often referred to as the candy cane limb, can accumulate food and secretions, causing stasis, distention, and pressure-related symptoms [7]. Several preventive techniques have been advocated, including ensuring the afferent limb is kept short, ideally no longer than 1-2 cm, and oriented straight to minimize redundancy [8]. Routine use of intra-operative endoscopy or passage of a nasogastric or orogastric tube can help verify the flow of contents and detect anatomical kinks or potential blind loops [9]. Additionally, robotic assistance may improve visualization and control during the construction of the anastomosis, reducing the risk of leaving behind a nonfunctional segment [6]. Standardizing intra-operative measurements and conducting a final inspection of the anastomotic site before closure are essential steps to prevent this complication [10].

When a candy cane limb remains, patients often endure vague symptoms that overlap with more common post-bypass conditions, leading to repeated investigations and delayed treatment [11]. Awareness of this anatomical pitfall and familiarity with diagnostic tools, contrast studies, cross-sectional imaging, and most definitely, upper endoscopy are crucial for timely identification. Once recognized, resection of the redundant limb reliably relieves symptoms, particularly when performed with minimally invasive or robotic techniques. 

## Case presentation

Case 1

**Figure 1:**
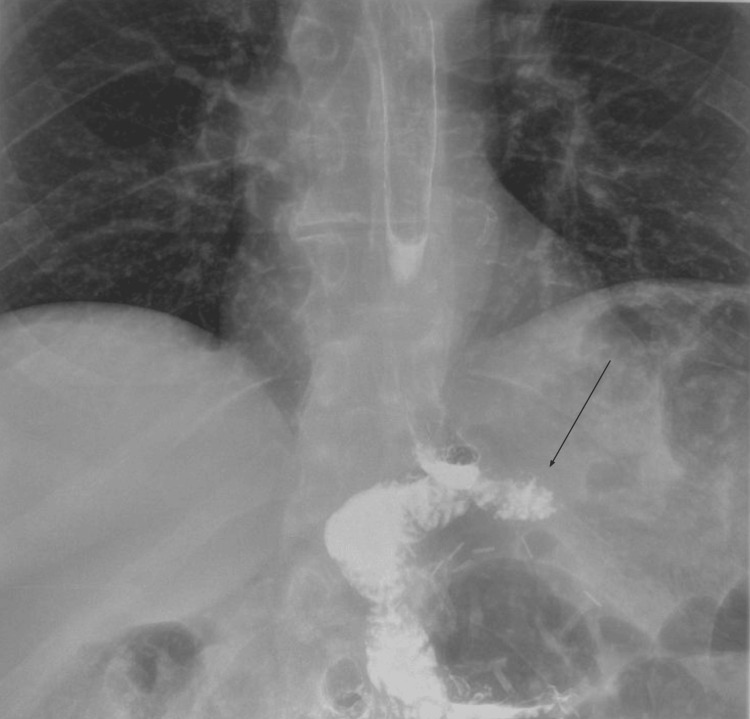
An upper GI contrast study with an arrow indicating the redundant candy cane limb GI: Gastrointestinal

A 45-year-old female patient with a BMI of 41.9 kg/m² underwent RYGB for the management of morbid obesity, type 2 diabetes, and obstructive sleep apnea. The procedure was performed using a standard antecolic, antegastric approach with a 150 cm Roux limb and an end-to-side, linear-stapled GJ. Five years later, she developed persistent epigastric pain, bloating, and nausea, especially after meals. An upper gastrointestinal contrast study suggested a blind-ending segment at the anastomotic site (Figure 1).

**Figure 2:**
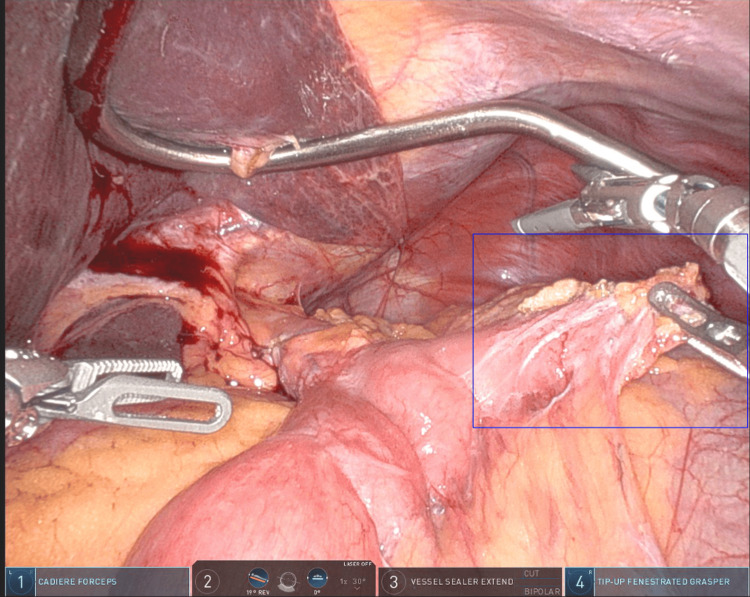
Intra-operative view, the blue box outlines the redundant afferent (candy cane) limb

Upper endoscopy confirmed a redundant blind limb just proximal to the GJ, consistent with CCS. She underwent a robotic-assisted revision involving resection of the blind afferent limb and re-creation of the anastomosis (Figure 2).

The patient's postoperative recovery was uncomplicated, and she experienced complete resolution of her symptoms.

Case 2

**Figure 3:**
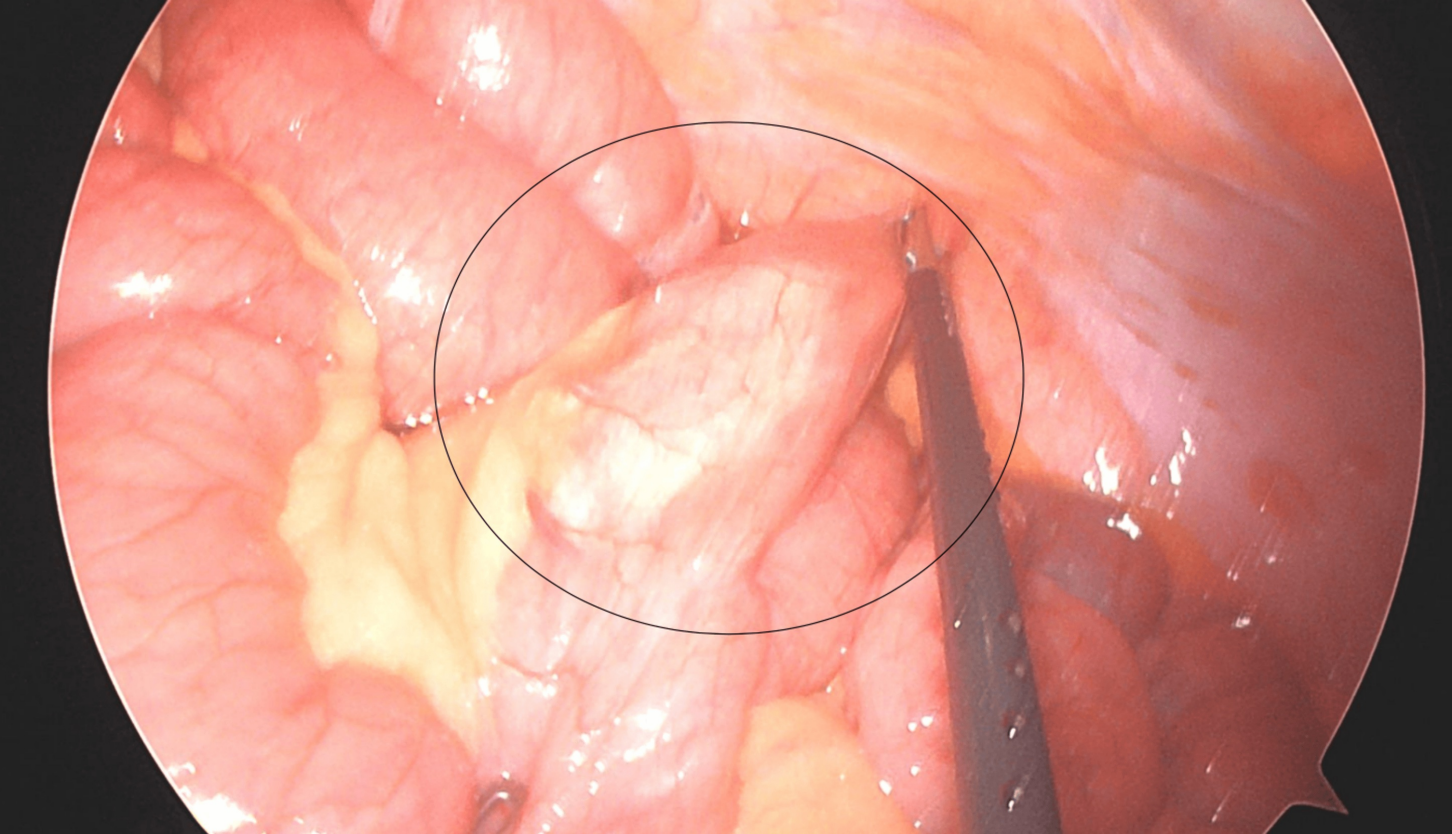
Intra-operative photograph demonstrating the redundant afferent (candy cane) limb

A 36-year-old female patient with a preoperative BMI of 60.8 kg/m² and a medical history of gastroesophageal reflux disease, infertility, and obstructive sleep apnea underwent RYGB. The Roux limb was constructed in an antecolic, antegastric fashion, and the GJ was created using a linear-stapled, side-to-side technique. Six years after the surgery, she developed severe episodic abdominal pain, nausea, and had limited oral intake. CT revealed a narrowing of the superior mesenteric vein and suggested internal herniation. Diagnostic laparoscopy revealed a redundant Roux limb contributing to functional obstruction (Figure 3).

Robotic-assisted revision with resection of the redundant limb was performed. Her postoperative course was uneventful, and she remained symptom-free on follow-up.

Case 3

**Figure 4:**
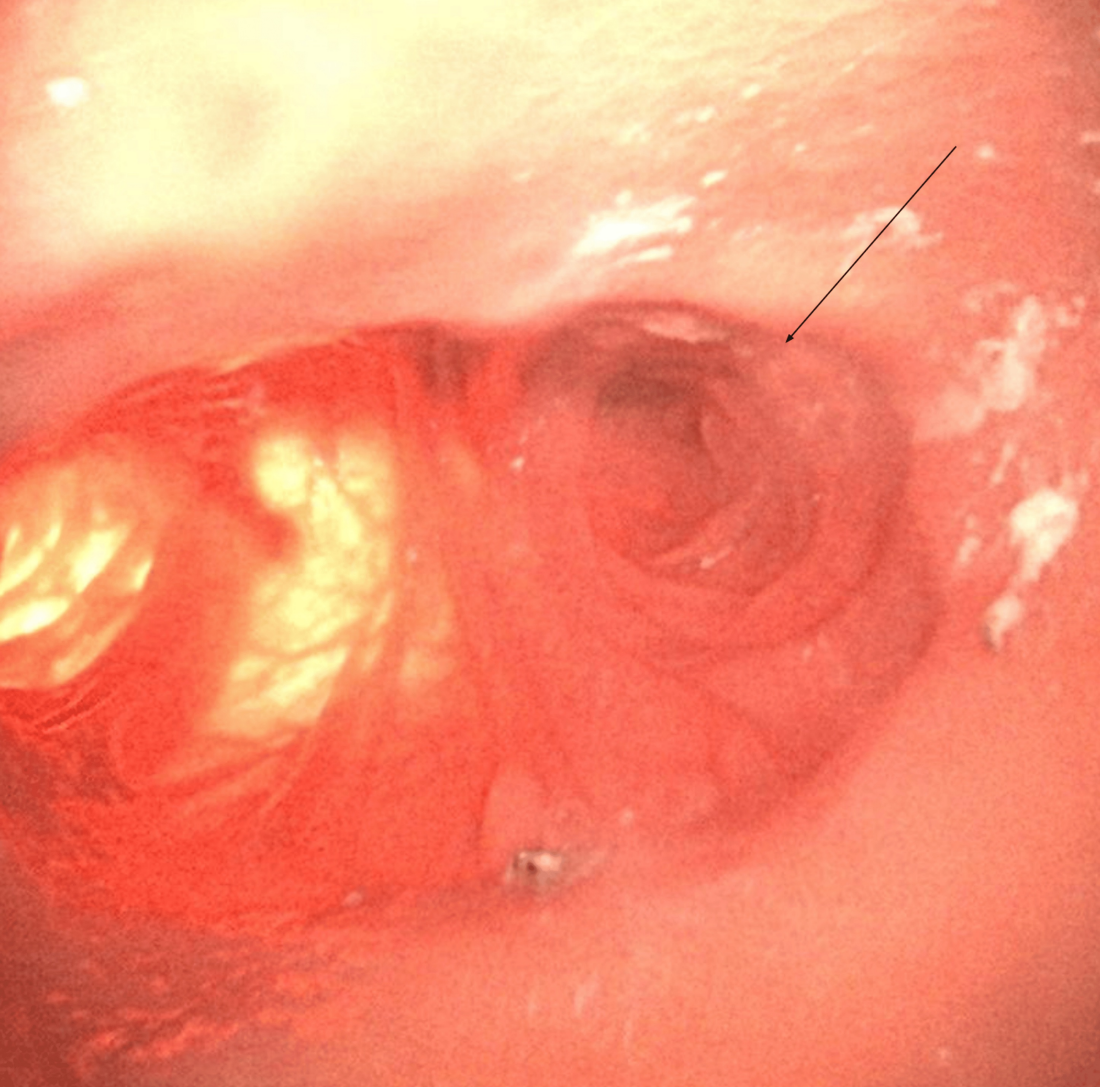
Intra-operative endoscopic view with the arrow marking the blind limb (the gastrojejunostomy is visible to the left)

A 56-year-old male patient with a BMI of 42.1 kg/m² and an extensive medical history including coronary artery disease, obstructive sleep apnea, and chronic lymphocytic leukemia underwent RYGB. The surgery involved a linear-stapled, side-to-side GJ and a 150 cm antecolic Roux limb. Several years later, he reported postprandial epigastric discomfort, nausea, and bloating. Imaging and upper endoscopy revealed a long, dilated afferent limb segment consistent with CCS (Figure 4).

**Figure 5:**
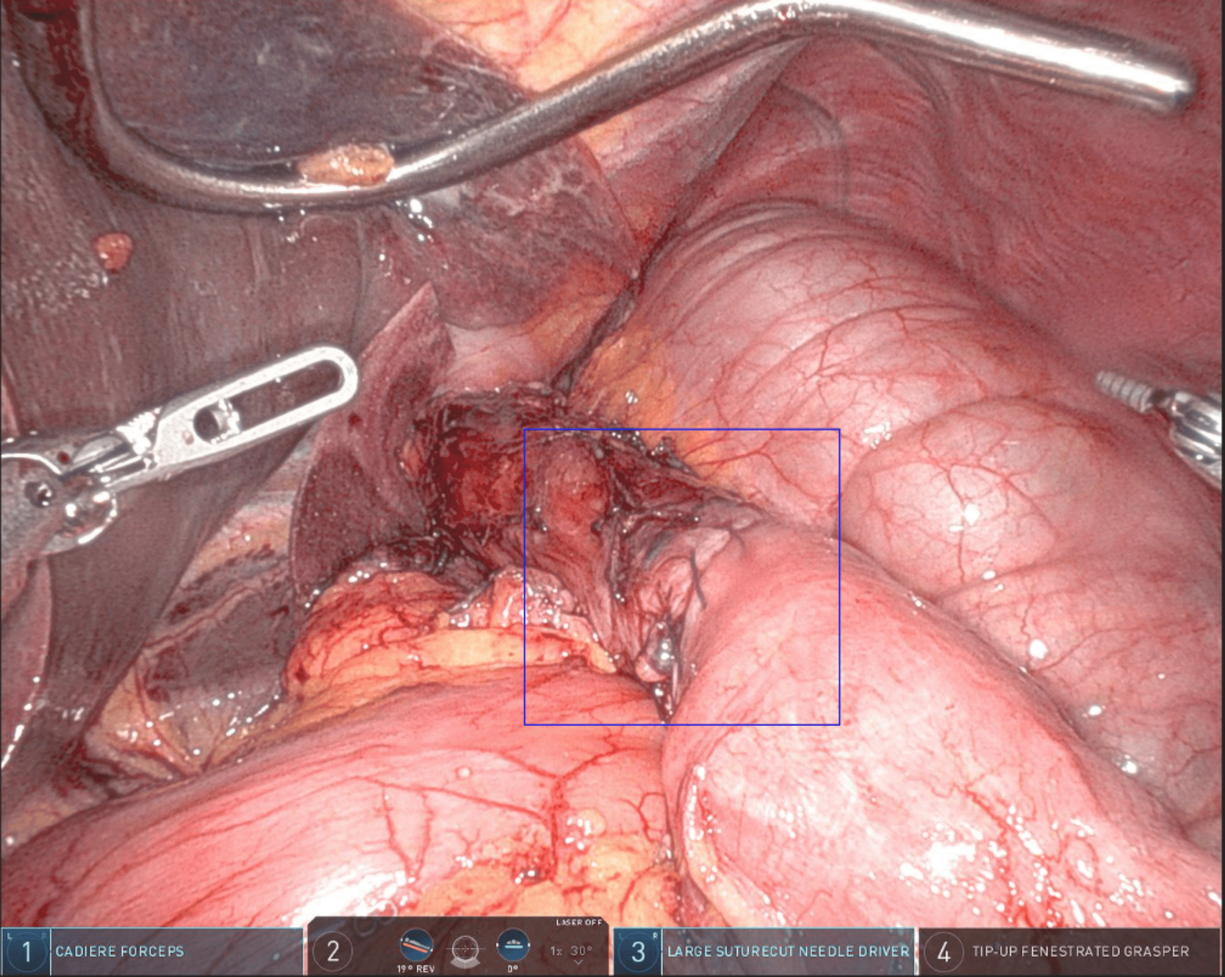
Post-revision view of the gastrojejunostomy after resection of the redundant limb and reconstruction of the anastomosis

He underwent a robotic revision with resection of the redundant limb (Figure 5).

His postoperative course was complicated by encephalopathy due to medication errors, but he was stabilized with supportive care. He subsequently reported marked improvement in gastrointestinal symptoms and maintained good dietary intake.

Case 4

**Figure 6:**
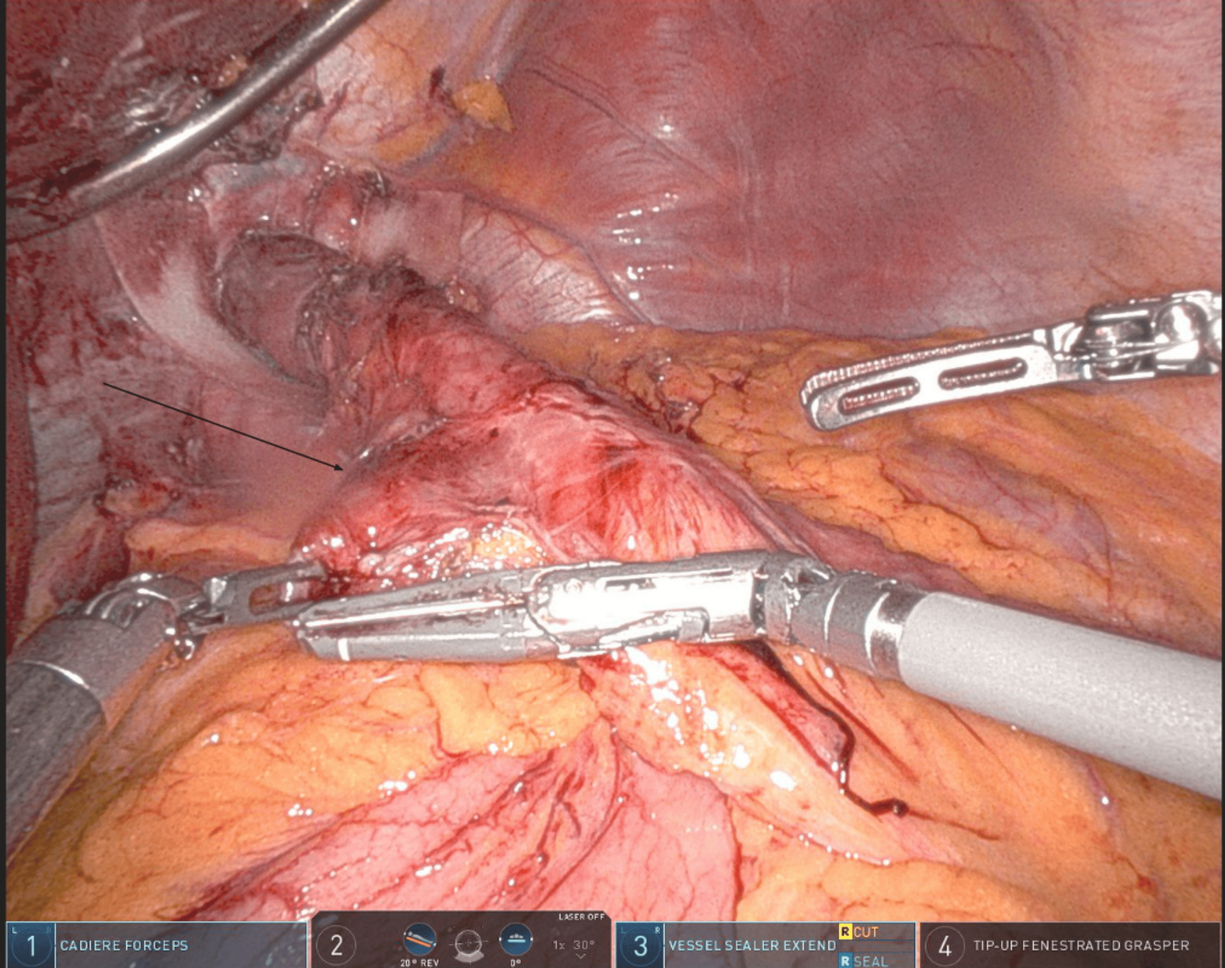
Intra-operative photograph with the black arrow identifying the redundant afferent (candy cane) limb

A 64-year-old female patient with a BMI of 34.2 kg/m² underwent robotic-assisted RYGB with simultaneous hiatal hernia repair to address severe gastroesophageal reflux, gastroparesis, and type 2 diabetes. The Roux limb was measured to 125 cm and positioned antecolically. A linear-stapled GJ was constructed. She developed progressive dysphagia, nausea, and vomiting within a month. Endoscopy confirmed a long afferent limb extending from the GJ, consistent with CCS. She underwent a robotic revision with resection of the redundant segment and reconfiguration of the anastomosis (Figure 6).

Her early recovery was complicated by transient edema causing dysphagia, necessitating hospital readmission. With conservative management, her symptoms resolved, and she achieved satisfactory nutritional intake with stable weight maintenance at follow-up.

The following table (Table 1) summarizes the key data for each case.

**Table 1:** Comparative clinical and surgical characteristics of four patients who developed CCS after Roux-en-Y gastric bypass CCS: Candy cane syndrome; UGI: Upper Gastrointestinal; GJ: Gastrojejunostomy

Metric	Case 1	Case 2	Case 3	Case 4
Age (yrs)	45	36	56	64
Sex	Female	Female	Male	Female
Pre-operative BMI (kg/m²)	41.9	60.8	42.1	34.2
Roux limb length (cm)	150	150	150	125
GJ technique	Linear-stapled end-to-side	Linear-stapled side-to-side	Linear-stapled side-to-side	Linear-stapled end-to-side
Interval to CCS (yrs)	5	6	4	0.1 (≈1 month)
Key presenting symptoms	Epigastric pain, bloating, nausea	Abdominal pain, nausea, poor intake	Post-prandial pain, bloating	Dysphagia, vomiting, nausea
Study confirming CCS	UGI + endoscopy	Diagnostic laparoscopy	UGI + endoscopy	Endoscopy
Revision approach	Robotic resection + new GJ	Robotic resection	Robotic resection	Robotic resection + GJ revision
Early complications	None	None	Transient encephalopathy (medication)	Transient edema causing dysphagia
Outcome at follow-up	Complete symptom resolution	Complete symptom resolution	Marked improvement, stable intake	Full symptom resolution, stable nutrition

These include the demographic profile, original bypass configuration, interval to symptom onset, presenting features, modality that confirmed the redundant afferent limb, revision strategy, early postoperative events, and clinical outcome. All gastrojejunostomies were created with a linear-stapled technique; differences in the “end-to-side” versus “side-to-side” approaches reflect the surgeon's preference at the index operation. The interval to the CCS ranged from 0.1 years (approximately one month) to six years, illustrating the variable latency of this complication. Revision was performed robotically in every patient, with complete or marked symptom relief at follow-up. Early complications were limited to transient, conservatively managed events in two cases (post-operative encephalopathy related to polypharmacy in Case 3 and temporary edema-induced dysphagia in Case 4). This side-by-side comparison highlights both the heterogeneous presentation of CCS and the consistent success of targeted limb resection.

## Discussion

CCS is a late postoperative complication of RYGB in which a redundant afferent Roux limb adjacent to the GJ forms a blind pouch [2]. This segment accumulates ingested material and secretions, producing symptoms such as postprandial epigastric pain, nausea, vomiting, dysphagia, bloating, and early satiety [12]. Though uncommon, it remains clinically significant and often underrecognized.

The construction of the Roux limb and GJ anastomosis in RYGB can be performed using different techniques [9]. The anastomosis may be hand-sewn, linear stapled, or circular stapled [13]. Linear staplers are favored for speed and lower stricture rates, while circular staplers create a uniform opening but may require a larger incision and carry a slightly higher risk of narrowing [13,14]. The choice of technique should prioritize precision and careful orientation to minimize unnecessary limb length and prevent the formation of a blind afferent segment.

**Figure 7:**
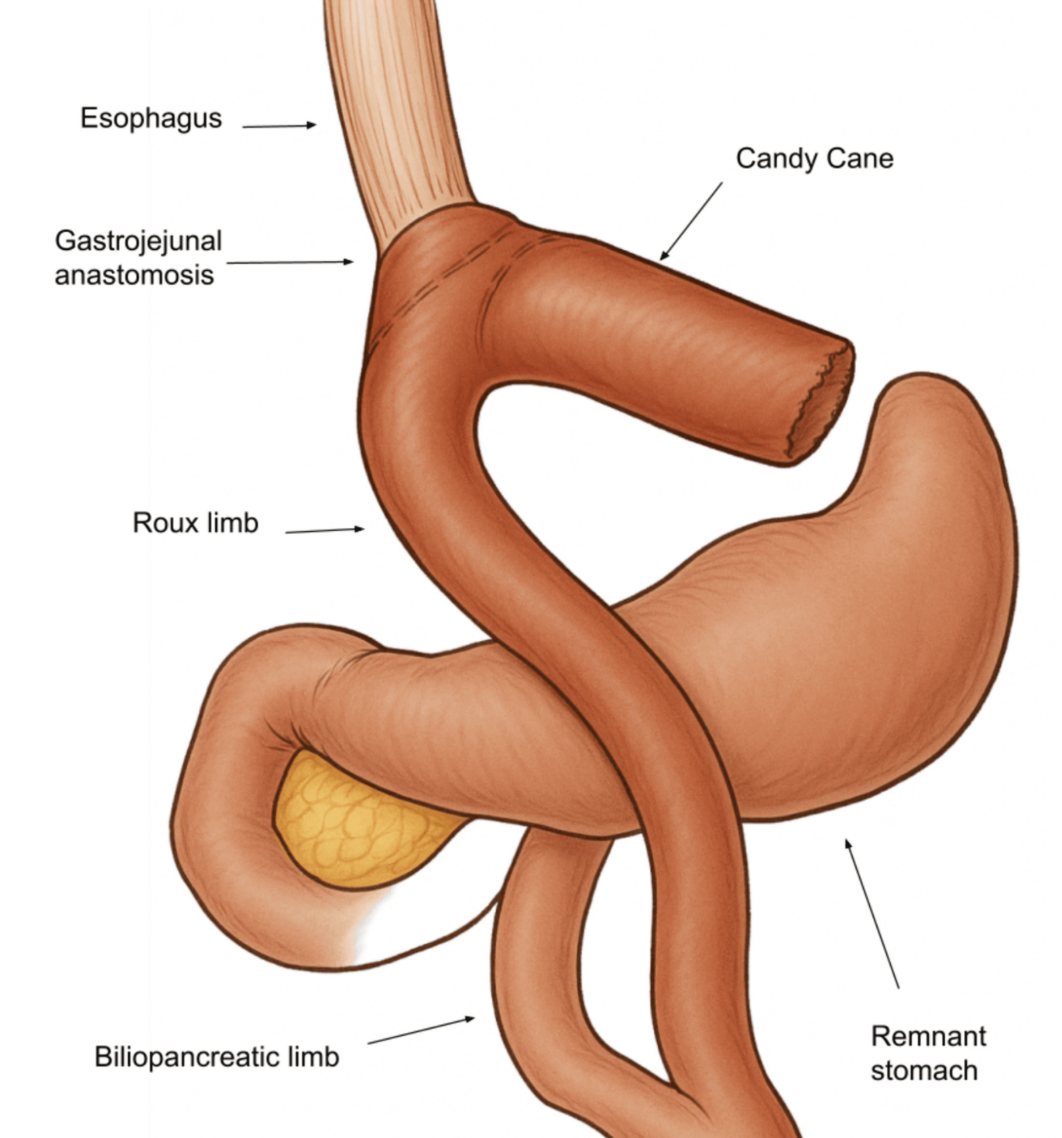
Anatomical illustration of candy cane syndrome showing a redundant afferent limb extending from the gastrojejunostomy Illustration created by Dr. Mena Louis and enhanced with the help of ChatGPT 4.1 (OpenAI, California, US)

CCS arises when the proximal limb is left too long (Figure 7), often due to visualization limitations or a desire to create a tension-free anastomosis [8].

Preventive strategies include trimming the afferent limb to 3-5 cm, ensuring the limb is not twisted, and confirming orientation with intraoperative endoscopy [7]. Attention to limb alignment and anatomical landmarks during the primary operation reduces the likelihood of this complication.

Pathophysiologically, symptoms result from food stasis and mechanical irritation within the blind limb. The incidence has been estimated at 1-4%, though it is likely underreported [12]. The symptoms of CCS overlap with other RYGB complications, making diagnosis challenging. Differential diagnoses include internal hernia, small bowel obstruction, anastomotic stricture, marginal ulcer, gastrogastric fistula, bile reflux, dumping syndrome, and delayed gastric emptying.

A thorough diagnostic workup begins with clinical history and physical examination focused on symptom timing and relation to food intake. Imaging modalities such as an upper gastrointestinal series may show a blind segment or delayed contrast passage [15]. Cross-sectional imaging, including CT or CT enterography, can help rule out internal hernias or obstruction. However, upper endoscopy remains the most effective diagnostic tool, as it allows direct visualization of the redundant limb and assessment of the anastomosis [7]. If imaging is inconclusive but suspicion remains, diagnostic laparoscopy can confirm the diagnosis and allow for immediate surgical correction.

All four patients in this case series presented with characteristic symptoms following RYGB, though the timeline and severity varied. In each case, initial workups were either non-diagnostic or suggested alternate causes, delaying the identification of CCS. Endoscopy ultimately confirmed the diagnosis in each case. Each patient underwent robotic-assisted revision involving resection of the redundant limb and reconstruction of the GJ. In all cases, symptoms resolved after revision, demonstrating the effectiveness of this approach.

Robotic-assisted revisional surgery for CCS provides several advantages compared to laparoscopic and open approaches, including enhanced three-dimensional visualization, improved dexterity, and greater precision in dissecting scarred or altered anatomy. These benefits can lead to safer resection of redundant limbs and reconstruction of the anastomosis, with a lower risk of injury to adjacent structures. Compared to open surgery, robotic approaches are associated with less pain, shorter hospital stays, and faster recovery, though drawbacks include higher costs and longer operative times. This case series is notable for its exclusive use of robotic revision, offering clear educational value with standardized techniques, detailed intraoperative images, and a practical diagnostic algorithm. 

Surgical technique during both primary and revisional operations plays a critical role in outcomes. Intraoperative endoscopy is highly useful for confirming proper orientation and limb length. Ensuring the afferent limb does not extend more than a few centimeters from the anastomosis, maintaining a straight Roux limb configuration, and avoiding torsion all contribute to long-term symptom-free outcomes.

Complications after revision are rare but must be monitored. In our series, one patient developed postoperative dysphagia due to edema, requiring brief hospitalization. Another experienced transient encephalopathy due to polypharmacy. Both were managed conservatively with full recovery. These cases stress the importance of individualized postoperative care, especially in complex patients.

## Conclusions

CCS is a preventable anatomical complication that can emerge months to years after RYGB, producing postprandial pain, nausea, dysphagia, or vomiting when a blind afferent limb is left too long at the GJ. In this four-patient series, symptoms resolved or markedly improved after robotic resection of the redundant segment, affirming surgical revision as the most reliable remedy once the diagnosis is confirmed. Early recognition relies on maintaining the condition in the differential for persistent upper-gastrointestinal complaints and employing targeted studies. such as contrast swallow, cross-sectional imaging, and, most decisively, endoscopy. Preventive measures during the index operation, like trimming the afferent limb, careful limb orientation, and intraoperative endoscopic inspection, reduce the likelihood of creating a blind pouch and its associated morbidity. Regular postoperative follow-up, a high index of suspicion, and readiness to intervene surgically when conservative evaluation reveals a redundant limb together ensure optimal outcomes for patients who undergo gastric bypass surgery.
